# Physical Disturbance Reduces Cyanobacterial Relative Abundance and Substrate Metabolism Potential of Biological Soil Crusts on a Gold Mine Tailing of Central China

**DOI:** 10.3389/fmicb.2022.811039

**Published:** 2022-04-06

**Authors:** Jingshang Xiao, Shubin Lan, Zulin Zhang, Lie Yang, Long Qian, Ling Xia, Shaoxian Song, María E. Farías, Rosa María Torres, Li Wu

**Affiliations:** ^1^School of Resources and Environmental Engineering, Wuhan University of Technology, Wuhan, China; ^2^Key Laboratory of Algal Biology, Institute of Hydrobiology, Chinese Academy of Sciences, Wuhan, China; ^3^The James Hutton Institute, Aberdeen, United Kingdom; ^4^Laboratorio de Investigaciones Microbiológicas de Lagunas Andinas (LIMLA), Planta Piloto de Procesos Industriales Microbiológicos (PROIMI), Centro Científico Tecnológico (CCT), Consejo Nacional de Investigaciones Científicas y Técnicas, San Miguel de Tucumán, Argentina; ^5^CETMIC- CONICET- CCT La Plata, Comisión de Investigaciones Científicas de la Provincia de Buenos Aires (CICBA), La Plata, Argentina

**Keywords:** mine tailing, physical disturbances, biological soil crusts, enzyme activity, nutrient content, bacterial community

## Abstract

As the critical ecological engineers, biological soil crusts (biocrusts) are considered to play essential roles in improving substrate conditions during ecological rehabilitation processes. Physical disturbance, however, often leads to the degradation of biocrusts, and it remains unclear how the physical disturbance affects biocrust microorganisms and their related metabolism. In this study, the photosynthetic biomass (indicated by chlorophyll *a*), nutrients, enzyme activities, and bacterial communities of biocrusts were investigated in a gold mine tailing of Central China to evaluate the impact of physical disturbance on biocrusts during the rehabilitation process of gold mine tailings. The results show that physical disturbance significantly reduced the photosynthetic biomass, nutrient contents (organic carbon, ammonium nitrogen, nitrate nitrogen, and total phosphorus), and enzyme activities (β-glucosidase, sucrase, nitrogenase, neutral phosphatase, and urease) of biocrusts in the mine tailings. Furthermore, 16S rDNA sequencing showed that physical disturbance strongly changed the composition, structure, and interactions of the bacterial community, leading to a shift from a cyanobacteria dominated community to a heterotrophic bacteria (proteobacteria, actinobacteria, and acidobacteria) dominated community and a more complex bacterial network (higher complexity, nodes, and edges). Altogether, our results show that the biocrusts dominated by cyanobacteria could also develop in the tailings of humid region, and the dominants (e.g., *Microcoleus*) were the same as those from dryland biocrusts; nevertheless, physical disturbance significantly reduced cyanobacterial relative abundance in biocrusts. Based on our findings, we propose the future work on cyanobacterial inoculation (e.g., *Microcoleus*), which is expected to promote substrate metabolism and accumulation, ultimately accelerating the development of biocrusts and the subsequent ecological restoration of tailings.

## Introduction

With the development of the economy, the consumption of mineral resources continues to increase, posing a severe ecological threat of mine tailings on surrounding environments. The remediation of mine tailings has been extremely difficult because of the adverse physicochemical properties, including extreme pH conditions, low nutrient contents, lack of aggregate structure, and high toxicity of heavy metals (Cabala et al., 2011). All these adverse physicochemical properties strongly limit the development of substrate and subsequent colonization of higher vegetation (Ye et al., 2002; Huang et al., 2011). However, compared with higher plants, microorganisms are highly adaptable to harsh environmental extremes (Remon et al., 2005; Alsharif et al., 2020; Kakeh et al., 2021), and biological soil crusts (biocrusts) are considered to be the soil ecological engineers in mine tailings (Stewart et al., 2014; Gypser et al., 2016; Nyenda et al., 2019a,b).

Biocrusts are a topsoil layer formed by the cementation of cyanobacteria, lichen, moss, bacteria, fungi, and other organisms with soil particles (Belnap, 1995; Wu et al., 2014; Belnap and Büdel, 2016). They are widely found in global harsh environments including drylands (Belnap et al., 2001; Wu et al., 2013), polar regions (Rippin et al., 2018), and mine tailings (Nyenda et al., 2019a) and account for a large proportion of global land surface (Rodriguez-Caballero et al., 2018; Kakeh et al., 2020). Their functions, including playing an important role in improving soil nutrients and water conditions (Jiang et al., 2018; He et al., 2019), maintaining topsoil stability (Belnap, 2003), and facilitating the succession of vascular plants (Lan et al., 2014b), are considered critical in the ecological restoration of local fragile ecosystems.

Biocrusts are vulnerable and sensitive to physical disturbance, such as vehicle traffic, trampling, plowing, grazing, and mining (Langhans et al., 2010; Hagemann et al., 2017) although biocrusts are strongly resistant to water and wind erosion. Physical disturbance could lead to reverse succession and even destruction of biocrusts (Darby et al., 2010; Ferrenberg et al., 2015). At small scales, physical disturbance destroys the structure of biocrusts and significantly reduces the ability of resisting wind (Munkhtsetseg et al., 2017; Gao et al., 2020; Papatheodorou et al., 2020). It is reported that the restoration of biocrusts would take more than 10 years after physical disturbance (Dojani et al., 2011), indicating that physical disturbance is an important factor restricting the development of biocrusts. At large scales, physical disturbance causes the reduction of biocrust coverage, which additionally affects the global soil biogeochemical process (Steven et al., 2014; Ferrenberg et al., 2015; Yang et al., 2018; Lafuente et al., 2020).

As primary colonizers, biocrusts can effectively improve the fertility and texture of substrate and, therefore, accelerate the following succession of vegetation (Lan et al., 2014a; Gypser et al., 2016; Nyenda et al., 2019a). However, in mine tailing areas, physical disturbance is a severe factor that threatens biocrust formation and long-term stability (Levi et al., 2021). To date, although the effects of physical disturbance on biocrusts are widely investigated in dryland, comparatively little attention is given to the effects of physical disturbance on biocrust development on mine tailings, particularly the shifts in microbial community interactions and the related material metabolism during the ecological rehabilitation processes. Although biocrusts can reform on the substrate of mine tailings after physical disturbance, the response and resilience of cyanobacteria (e.g., *Microcoleus*) and other biocrust bacteria to degrees of physical disturbance is still unknown. Understanding the threat and effect mechanism of physical disturbance on biocrusts will help us in remediating and managing mine tailings.

In this study, biocrusts with varying degrees of physical disturbance were collected in a series of gold mine tailings of Central China, and the nutrient conditions, enzyme activities, structure, and interactions of the bacterial community are investigated to reveal the effects and mechanism of physical disturbance on biocrust development during the rehabilitation process of gold mine tailings. In particular, this study explores the potential mechanism of biocrust bacterial community shift and interactions, which drive the improvement of substrate conditions in mine tailings. We hypothesized that physical disturbance would (1) decrease biocrust nutrient contents and enzyme activities, and (2) lead to distinct biocrust bacterial communities after disturbance. The results will provide a theory basis for the ecological restoration and management of mine tailings and are also helpful for understanding the impacts of physical disturbances on fragile ecosystems.

## Materials and Methods

### Study Site

**FIGURE 1:**
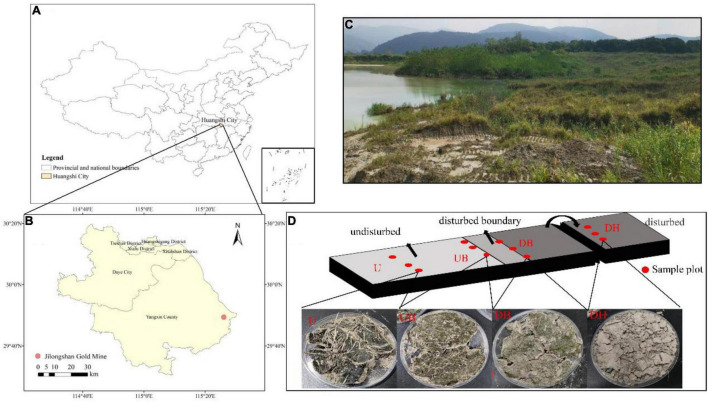
Location of the gold tailings **(A,B)**, landscapes of the experimental area **(C)**, the diagram of the position where the samples were collected and a sample image of different disturbance areas **(D)**. DH- Heavily disturbed area; DB- disturbed boundary area; UB- Undisturbed boundary area approximately 1–1.5 m away from the disturbed boundary area; U-Undisturbed area.

The study site is located in gold and copper mine tailings in Daye City, Hubei Province, Central China (Figures 1A–C). Mean annual temperature and rainfall are 16.8°C and 1389.6 mm, respectively. This area is a subtropical monsoon region. Most of the annual precipitation occurs during April through July. In this study, the mine tailings have been sealed for 15 years. Last year, some areas were mechanically excavated due to production needs.

### Field Sampling and Experimental Setup

In October 2020, a total of four sample areas with different biocrust disturbance were selected in the mine tailings. According to the degree of physical disturbance, the biocrust samples collected from these four areas were designated as a heavily disturbed area (DH), disturbed boundary area (DB), undisturbed boundary area (UB), and undisturbed area (U). In the heavily disturbed area, the tailing dune was completely disturbed and replied up approximately 1 year before the experiment. Therefore, the biocrust samples (DH) collected from this area were newly formed within 1 year after the disturbance. DB samples were collected at the boundary of the disturbed and undisturbed areas, caused by trampling by people. UB samples were collected in the undisturbed area in a distance approximately 1–1.5 m from the disturbance boundary. U samples were collected in a completely undisturbed area (approximately 15 years). The diagram of sampling positions is shown in Figure 1D, and the other detailed characteristics of the sampling plots and biocrusts are shown in Table 1. The collected biocrust samples were placed in a sterile plastic plate using a sterilized spatula (Figure 1D) and quickly brought back to the laboratory. Three biocrust samples from each disturbed area were collected after removing roots and brushing off the bottom soil, generating 12 samples in total.

**TABLE 1:** Basic characteristics of experimental plots.

Plot number	Disturbed time	Biocrust color	Developed level of biocrusts	Degree of disturbance
DH	1 Year ago	Gray	Cyanobacteria (disturbed 1 year ago)	Heavy (mechanical overturn)
DB	1 Year ago	Gray-green	Cyanobacteria (disturbed boundary)	Slight (people trample)
UB	0 Year	Green	Cyanobacteria (undisturbed boundary)	None
U	0 Year	Black	Cyanobacteria; Moss (undisturbed)	None

### Analysis of Biocrust Physicochemical Properties

Ten physicochemical parameters were determined for the collected samples, including pH, EC, exopolysaccharides (EPS), chlorophyll *a*, scytonemin, nitrate nitrogen (NO_3_-N), ammonium nitrogen (NH_4_-N), organic carbon (OC), total phosphorus (TP), and soil particle distribution (PSD). The biocrust samples were suspended in ultrapure water (soil: solution = 1:10), and pH value and EC value were measured with a pH electrode potentiometer (PHBJ-260F INESA, China) and a conductivity meter (DDSJ-308F INESA, China), respectively. Chlorophyll *a* and scytonemin were extracted from the biocrust samples using acetone, and the absorbance of the extracts was determined at 663, 490, and 384 nm. Finally, the content of chlorophyll *a* and scytonemin were calculated by the three-color formula (Garcia-Pichel and Castenholz, 1991). Eight ml ultrapure water was used to extract EPS from each biocrust sample in a water bath (80°C) for 2 h and then centrifuged at 5,000 × g for 10 min. The phenol sulfuric acid method was used to determine the EPS content (Dubois et al., 1956). NO_3_-N and NH_4_-N were determined using the phenol disulfonic acid colorimetric method (Doane and Horwath, 2003) and indophenol blue colorimetry method (Wang et al., 2015), respectively. OC was determined using the H_2_SO_4_-K_2_Cr_2_O_7_ oxidation method (Nelson, 1996). TP was determined using a fully automatic discontinuous analyzer (Smartchem140 AMS-Alliance, Italia). To measure the soil particle distribution, samples were pretreated in H_2_O_2_ solution (30%, w/w) to remove organic matter and then dispersed by adding sodium hexametaphosphate and finally analyzed using a laser particle size analyzer (Mastersizer 2000, England). The compositions and contents of biocrusts elements were determined using an ICP-OES (Leeman, Mason, United States). Before determination, all the samples were digested in aqua regia at 200°C, and then the volume was adjusted to 50 ml for injection. The selected wavelengths for different elements were as follow: Al (396.152 nm), Mg (285.213 nm), Mn (257.610 nm), Fe (259.940 nm), Ca (317.933 nm), Na (589.592 nm), S (180.731 nm), Ti (334.941 nm), and K (766.491 nm). Reagent-matched standards were used for element analysis in each digestion method (Marguí et al., 2005).

### Determination of Biocrust Enzyme Activities

Seven enzyme activities linked to carbon (α-glucosidase, β-glucosidase, and sucrase), nitrogen (neutral protease, urease, and nitrogenase), and phosphorus (neutral phosphatase) cycling were analyzed. The enzyme activities, including α-glucosidase, β-glucosidase, sucrose, neutral protease, urease, nitrogenase, neutral phosphatase, peroxidase, and polyphenol oxidase were measured using soil enzyme assay kits (Boxbio, Beijing, China; Solarbio, Beijing, China; Jingmei biotechnology, Jiangsu, China) according to the manufacturer’s protocols.

### DNA Extraction, Amplification, and Sequencing

DNA was extracted from each biocrust sample using the E.Z.N.A.® soil kit (Omega Bio-Tek, Norcross, GA, United States), and then the NanoDrop2000 spectrophotometer was used to detect the concentration and purity of the extracted DNA. The V3-V4 hypervariable regions of the bacteria 16S rDNA gene were amplified with primers 338F (5′-ACTCCTACGGGAGGCAGCAG-3′) and 806R (5′-GGACTACHVGGGTWTCTAAT-3′) by the thermocycler PCR system (GeneAmp 9700, ABI, United States). PCR reactions were performed in triplicate in a 20 μL mixture containing 4 μL of 5 × FastPfu Buffer, 2 μL of 2.5 mM dNTPs, 0.8 μL of each primer (5 μM), 0.4 μL of FastPfu Polymerase, and 10 ng of template DNA. The resulting PCR products were extracted from a 2% agarose gel and further purified using the AxyPrep DNA Gel Extraction Kit (Axygen Biosciences, Union City, CA, United States) and quantified using QuantiFluor™-ST (Promega, United States) (Zhang et al., 2018). According to the standard operating procedures of the Illumina MiSeq platform (Illumina, San Diego, United States), the purified amplified fragments were subjected to PE library construction. Sequencing was performed using the MiSeq PE3000 platform of Illumina (Shanghai Majorbio Bio-Pharm Technology Co., Ltd). The raw reads were deposited into the NCBI Sequence Read Archive (SRA) database (Accession Number: SRP 297388). Sequencing numbers of each sample were rarified to the sample with the minimum number of 18,411 reads. The detail information about Illumina MiSeq sequencing data processing is provided in Supplementary Method.

Amplicon sequence variants (ASVs) were clustered with a 100% similarity cutoff, and chimeric sequences were identified and removed using DADA2. The taxonomy of each 16S rDNA gene sequence was analyzed by RDP Classifier algorithm^1^ against the Silva (Silva138) 16S rDNA database using a confidence threshold of 70%. The alpha diversity indices (Sob, ACE, Chao1, Shannon, Simpson) were calculated based on the ASVs.

### Network Construction and Analysis

Networkx software and random matrix theory were used to construct cooccurrence networks, including data collection, data transformation, pairwise similarity matrix calculation, and the adjacent matrix determination. Detailed information on the network construction is provided in Supplementary Method. A threshold of 0.999 was used to construct the bacterial networks. Spearman’s correlation coefficient was used to calculate the interaction between two microbes. The size of the node is proportional to its connectivity, and the color represents the bacterial phyla. The positive correlation between the nodes is represented by pink, and the negative correlation is represented by green. The visualization of networks was carried out in Gephi (version 0.9.2)^2^.

### Statistical Analysis

All the variations of soil physicochemical characteristics, enzyme activity, α-diversity, and element composition were analyzed using one-way ANOVA at 95%. The Kruskal–Wallis rank sum test was used to test the variation of bacterial community composition. The variation analysis was performed using SPSS (Version 22, IBM Corp, United States). A non-matrix multidimensional scaling (NMDS) plot following an analysis of similarity (ANOSIM) test based on Bray–Curtis similarity was performed in R with the Vegan package to visualize and assess the differences of bacterial community structure at the ASV level between different disturbed biocrusts.

## Results

### Physicochemical Properties and Elemental Composition of Biocrusts

Our results clearly show that physical disturbance affected the physicochemical properties of biocrusts (Table 2). Higher EC and pH values were found in the disturbed biocrusts (DH, DB; *P* < 0.05). Chlorophyll *a* content as the indication of photosynthetic biomass in biocrusts decreased as the disturbance degree increased (*P* < 0.05). In addition, other physicochemical properties, including Scytonemin, NO_3_-N, NH_4_-N, and OC also showed similar results to chlorophyll *a* (*P* < 0.05), and the highest total potassium content occurred in DH (*P* > 0.05). However, our results show that the degree of disturbance had no significant effect on EPS content in biocrusts (*P* > 0.05).

**TABLE 2:** Biocrust physiochemical properties (*n* = 3).

	DH	DB	UB	U
EC (μs/cm)	117.5 ± 11.88^a^	64.93 ± 14.54^b^	61.93 ± 3.61^b^	48.6 ± 4.98^b^
pH	8.61 ± 0.23^a^	7.56 ± 0.21^b^	7.10 ± 0.05^b^	7.37 ± 0.41^b^
Chl-a (μg/g)	2.09 ± 0.84^d^	3.78 ± 0.21^c^	7.74 ± 0.58^b^	8.99 ± 0.15^a^
Scytonemin (μg/g)	5.72 ± 3.16^d^	33.48 ± 4.08^c^	149.21 ± 4.57^b^	201.06 ± 5.73^a^
NO_3_-N (mg/kg)	9.74 ± 1.17^d^	14.38 ± 0.80^c^	21.19 ± 1.69^b^	24.34 ± 1.62^a^
NH_4_-N (mg/kg)	0.45 ± 0.04^d^	2.53 ± 0.01^c^	3.39 ± 0.03^b^	3.62 ± 0.01^a^
OC (g/kg)	8.00 ± 1.47^b^	13.24 ± 0.54^b^	22.97 ± 5.32^a^	25.57 ± 1.72^a^
EPS (mg/g)	1.30 ± 0.15^a^	1.40 ± 0.10^a^	1.32 ± 0.05^a^	1.27 ± 0.03^a^
TK (g/kg)	4.08 ± 0.31^a^	3.29 ± 0.25^b^	2.96 ± 0.21^b^	3.89 ± 0.23^a^
TP (g/kg)	0.20 ± 0.03^d^	0.45 ± 0.04^c^	0.63 ± 0.05^b^	0.75 ± 0.07^a^
Clay (<2 μm) (%)	9.82 ± 1.28^b^	9.11 ± 1.53^b^	15.84 ± 1.74^a^	16.63 ± 0.56^a^
Silt (2–20 μm) (%)	59.52 ± 3.13^b^	53.18 ± 2.43^c^	66.09 ± 2.31^a^	66.37 ± 2.01^a^
Sand (>20 μm) (%)	30.66 ± 4.40^a^	37.71 ± 0.90^a^	18.06 ± 3.93^b^	17.01 ± 2.51^b^

*Chl-a, Chlorophyll a; EC, electrical conductance; EPS, extracellular polysaccharide; NO_3_-N, nitrate nitrogen; NH_4_-N, ammonium nitrogen; OC, organic carbon; TK, total Kalium; TP, total phosphorus.*

*The letters indicate statistical differences in the results of analysis of variance between different tissues with a significant difference of P < 0.05.*

A total of 23 elements were detected in biocrusts with a Inductively Coupled Plasma Optical Emission Spectrometer (ICP-OES), and eight elements with relative higher contents are shown in Supplementary Table 1. There was no significant difference in the content of Na and S among different biocrust groups (*P* > 0.05). The content of Mg decreased with the increase of the disturbance degree (*P* < 0.05). The lowest content of Mn and Ca occurred in U (*P* < 0.05). Physical disturbance had the significant effect on soil particle distribution that higher clay and silt contents were found in undisturbed biocrusts (*P* < 0.05).

### Enzyme Activities of Biocrusts

**FIGURE 2:**
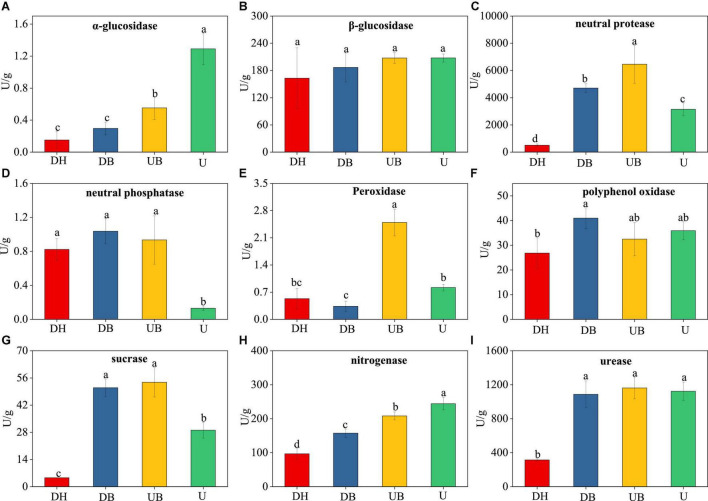
Changes in the enzyme activity of the biocrusts under different disturbance levels (*n* = 3). **(A)** α-glucosidase. **(B)** β-glucosidase. **(C)** neutral protease. **(D)** neutral phosphatase. **(E)** peroxidase. **(F)** polyphenol oxidase. **(G)** sucrase. **(H)** nitrogenase. **(I)** urease. Significant differences (*P* < 0.05) are marked by different letters.

Physical disturbances had significant effects on eight enzyme activities (Figure 2). The activities of α-glucosidase and nitrogenase decreased with increasing disturbance degree (Figures 2A,H). The lowest activities of urease and sucrase occurred in DH (Figures 2G,I). The lowest activity of neutral protease occurred in U (Figure 2D). The highest activities of neutral phosphatase and peroxidase were found in UB (Figures 2C,E).

### Species Composition, Diversity, and Richness of Bacterial Communities in Biocrusts

**FIGURE 3:**
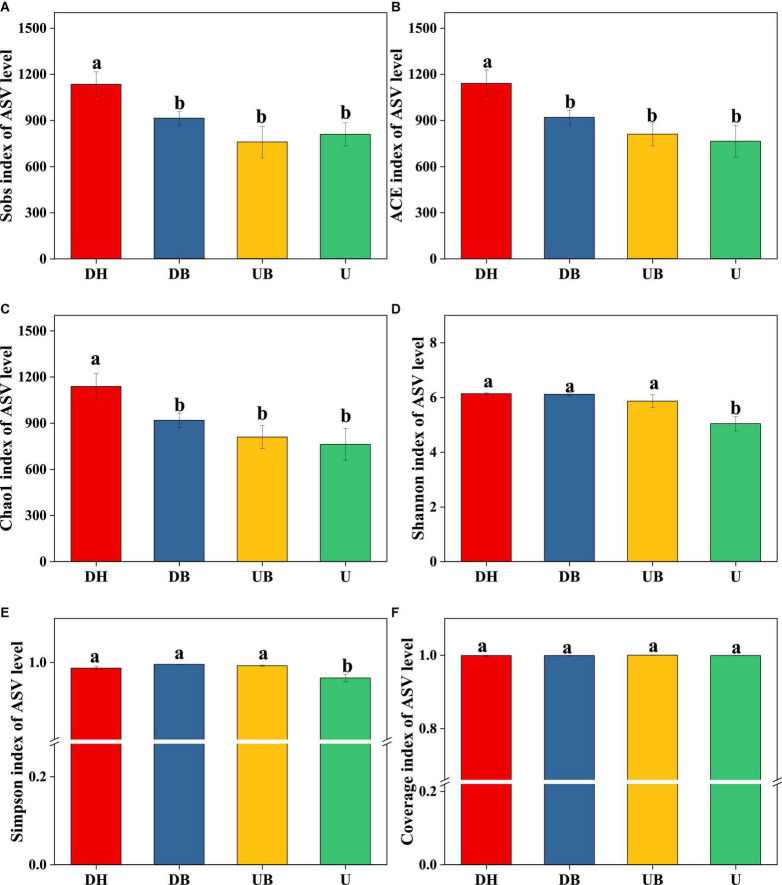
Bacterial α-diversity at different disturbance levels of biocrusts (*n* = 3). **(A)** Sobs index. **(B)** ACE index. **(C)** Chao1 index. **(D)** Shannon index. **(E)** Simpson index. **(F)** Coverage index. Significant differences (*P* < 0.05) are marked by different letters.

**FIGURE 4:**
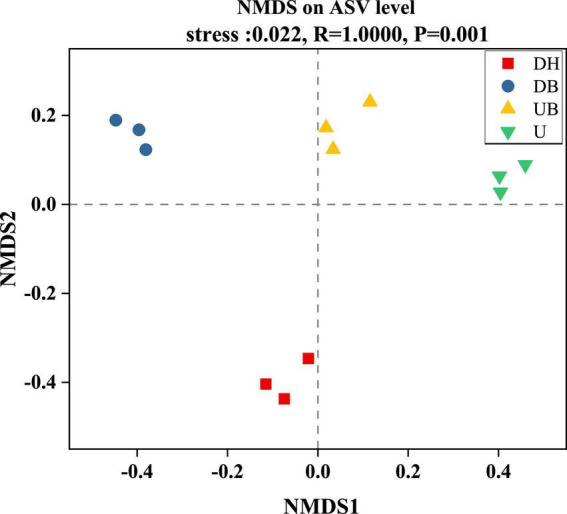
Non-metric multidimensional scaling of bacteria community structure between biocrusts at different disturbance levels.

All three α-diversity indices, Chao1, ACE, and Sobs, are richness estimates of a community. In this study, the Sobs index in DH was much higher than in the other three groups (Figure 3A, *P* < 0.05). Chao1 and ACE indices showed similar changes (Figures 3B,C), DH > DB > UB > U (*P* < 0.05). Shannon and Simpson diversity indices, the estimate of diversity of bacterial communities (Figures 3D,E), in U were much lower than in the other three groups (*P* < 0.05). According to the coverage index, the sequencing depth is adequate for this study (Figure 3F). Non-metric multidimensional scaling (NMDS) analysis showed that physical disturbance had a significant impact on the bacterial community structure of the biocrusts (*P* = 0.001). The biocrust samples with different disturbance degrees formed clearly delimited groups (Figure 4).

**FIGURE 5:**
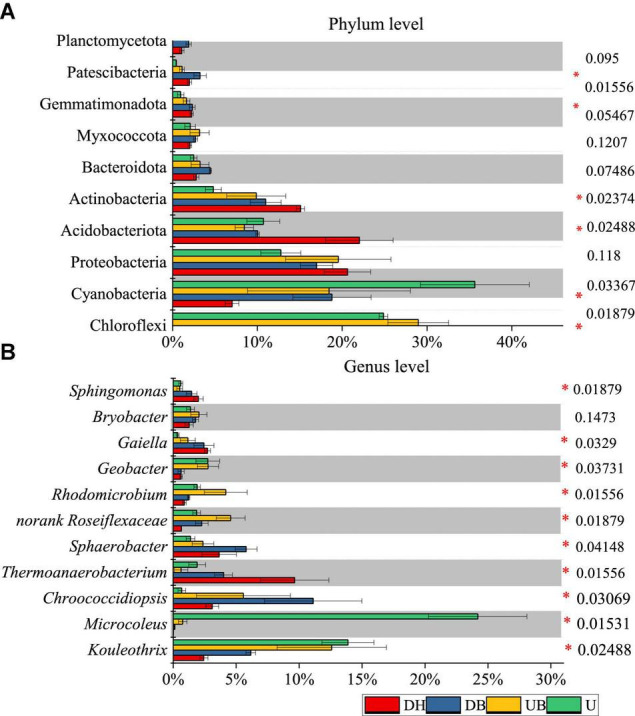
Average relative abundance of bacteria at phylum **(A)** and genus **(B)** level (*n* = 3). *P*-values are listed at right, * represents the significant differences at 0.05.

At the phylum level of bacteria, the relative abundant phyla across all samples were Chloroflexi, Cyanobacteria, Proteobacteria, Acidobacteriota, and Actinobacteria. The relative abundance of these five phyla together was more than 80% of total bacterial sequences (Figure 5A). Compared with the disturbed areas (DH, DB), higher relative abundances of Cyanobacteria and Chloroflexi and lower relative abundances of Actinobacteria were significantly found in UB and U (*P* < 0.05). In addition, the low-relative abundance phyla Bacteroidota, Myxococcota, Gemmatimonadota, and Patescibacteria were all detected in each biocrust sample. Planctomycetota was only found in disturbed samples (DH, DB). The relative abundance of Cyanobacteria in DH was 8%, whereas it was as high as 35% in U (Figure 5A).

At the genus level of bacteria, we observed significant differences between biocrust samples. *Microcoleus* was the most abundant genus, accounting for 24.19% of the total bacterial abundance in U (Figure 5B), much higher than the other genera (*P* < 0.05). The relative abundance of *Thermoanaerobacterium* was significantly higher in DH than in the other biocrust samples (*P* < 0.05). The relative abundance of *Kouleothrix* declined gradually with disturbance degrees from 13.88 (U) to 2.43% (DH).

### Correlation Networks of Bacterial Communities in Biocrusts

**FIGURE 6:**
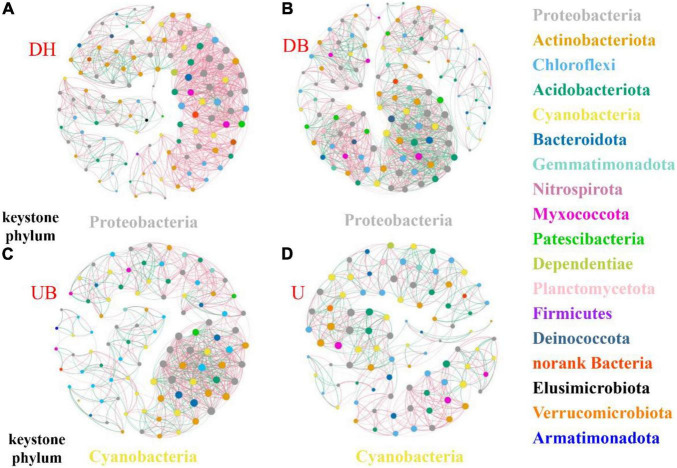
Co-occurrence networks of biocrusts bacterial communities in different disturbance biocrusts. **(A)** DH network. **(B)** DB network. **(C)** UB network. **(D)** U network. The nodes are colored by the phylum level. The size of each node is proportional to the node degree. The link between each pair of nodes represents positive (pink) and negative (green) correlation.

The network analysis showed that physical disturbance significantly impacted the topological properties of bacterial networks (Figure 6 and Table 3). The number of network nodes increased from 92 in U to 122 in DH. Disturbed (DH, DB) and undisturbed (UB, U) biocrusts showed significant differences in edges. The number of edges in the networks were 1,069 (DH), 1,159 (DB), 770 (UB), and 541 (U), respectively. Specifically, 868, 672, 462, and 319 positive edges were identified in the DH, DB, UB, and U networks, respectively, and negative edges numbering 201, 487, 308, and 222 were recorded correspondingly. The results showed that there were more positive edges than negative edges in all bacteria networks, and the proportion of positive edges was the highest in UB (81.2%), much higher than the other three networks, ranging from 58.0 to 60.0%.

**TABLE 3:** Network topological properties between different disturbance biocrusts.

Network properties	DH	DB	UB	U
Total nodes	120	122	100	92
Total edges	1,069	1,159	770	541
Negative edges (percentage)	201 (18.8)	487 (42.0)	308 (40.0)	222 (41.0)
Positive edges (percentage)	868 (81.2)	672 (58.0)	462 (60.0)	319 (59.0)
Average clustering coefficient	0.744	0.758	0.772	0.74
Average path distance	4.927	4.507	5.228	5.803
Modularity	0.569	0.606	0.567	0.634
Complexity	8.91	9.50	7.70	5.88

## Discussion

### Physical Disturbance Reduced the Photosynthetic Biomass and Nutrients in the Tailing Substrate

It is considered that the critical role of biocrusts on mine tailing remediation mainly relies on the improvement of the nutrient conditions of the tailing substrate, providing favorable conditions for the restoration of higher vegetation (Cabala et al., 2011; Huang et al., 2016). With the development of biocrusts, higher microbial biomass and their metabolic activities strongly improve topsoil conditions (Liu et al., 2012; Lan et al., 2013; Nyenda et al., 2019a). Chlorophyll *a* content is usually used to indicate the photosynthetic biomass in biocrusts and is positively related to the development of biocrusts (Lan et al., 2013, 2017; Chen et al., 2014). Our research shows that physical disturbance significantly reduced the chlorophyll a content. In addition, it was also found that scytonemin, an important sun-screening pigment in cyanobacteria (such as *Scytonema* and *Nostoc*), significantly decreased with the increase of the disturbance degree, and this result is highly consistent with the change of the biocrust color. The surface color of DH and DB was gray, and the undisturbed UB and U were black due to the large number of *Scytonema* distributed on the undisturbed plot surfaces. Broadly, it is found that high-intensity disturbance could destroy the original, better developed biocrusts, causing the reversal development of biocrusts from moss- to cyanobacteria-dominated (Belnap, 2006; Faist et al., 2017).

Soil nutrients are the material basis for the survival and development of organisms (Lan et al., 2015). Consistent with our initial hypothesis, physical disturbance (DH) significantly reduced soil nutrients (e.g., NO_3_-N, NH_4_-N, OC, and TP). The low N level and N accumulation rate in the tailings substrate are known as the main factors restricting the ecological restoration of mine tailings (Huang et al., 2011). Our research results show that, in the undisturbed biocrusts (UB, U), NO_3_-N content was approximately 21.19–24.34 mg/kg, and NH_4_-N content was 3.39–3.62 mg/kg, significantly higher than those in the disturbed biocrusts (DH, DB), providing direct evidence that physical disturbance significantly reduced the N accumulation in tailing substrate. Phosphorus (P) contents are key factors affecting diazotroph diversity and abundance (Cerna et al., 2009), moreover, P restriction could lead to changes in the bacteria community structure (Pushkareva et al., 2021). In this study, heterotrophic bacteria were dominant in DH (low P), and autotrophic bacteria were dominant in U (high P). Our results verify that biocrusts significantly improved the fertility of substrate in mine tailings, whereas destruction of biocrusts significantly reduces nutrients, which might greatly slow down the subsequent ecological restoration of vascular plants. OC always constitutes a significant fraction of biocrust nutrient content, and physical disturbance significantly reduced the content of OC in biocrusts, ranging from 25.57 (g/kg) in U to 8.00 (g/kg) in DH.

Compared with other types of soil, mine tailings have much higher metal levels, and the development and succession of biocrusts were significantly related to metal elements. Bowker et al. (2016) find that the distribution and development of moss crusts were positively correlated with the content of Mn, Mg, K, and Zn in the soil. K and Ca are not only the components of important compounds in photosynthetic cells, but they also play an active role in the physiological and metabolic activities of biocrusts (promoting enzyme synthesis and improving photosynthetic efficiency) (Bowker et al., 2016). In this study, the highest and lowest content of Mg was found in DH and UB, respectively (Supplementary Table 1). Physical disturbance destroyed the structure of the biocrusts, and Mg cannot be used by photosynthetic organisms and converted into chlorophyll. As ecological engineers, biocrusts always first colonize in bare sand and mine tailings with adverse soil conditions, and gradually improve soil fertility by microbial activities, therefore accelerating the rehabilitation of local ecosystem (Song et al., 2014).

In our research, it was found that physical disturbance had no significant difference on the EPS among different plots (Table 1). This result was different from biocrusts in dryland areas (Colica et al., 2014). It was revealed that the EPS content in stable biocrusts was significantly higher than that in disturbed biocrusts (Fick et al., 2020). Moreover, EPS content was approximately 1.3 mg/g in this study, which was relatively lower than that in dryland areas, ranging from 1.25 to 3.5 mg/g in global drylands (Chen et al., 2014; Rossi et al., 2018). Studies show that EPS play an important role in stabilizing sand surface (Whistler and Kirby, 2002) and resisting dryland stresses (Potts, 1994, 1999), especially in drought resistance (Rossi et al., 2012; Rossi and De Philippis, 2016). Our study area is in a humid zone, and water is no longer the main limiting factor, which may help explain why EPS content of biocrusts in our study stayed at a relative low level.

### Physical Disturbance Depressed Enzyme Activities of Biocrusts

Soil enzymes and microorganisms play an important role in regulating the material cycle in the soil; however, they are very sensitive to environmental changes (Skuji, nš and Burns, 1976; Zhang et al., 2020). This study found that physical disturbance significantly reduced most soil enzyme activities, including α-glucosidase, neutral phosphatase, sucrase, and urease (Figure 2), whereas physical disturbance had no effect on β-glucosidase. The α-glucosidase, sucrase, nitrogenase, urease, and neutral phosphatase are considered to be the crucial enzymes for soil C, N, and P metabolism (Li et al., 2019). The low enzyme activities after disturbance indicate a decline of C, N, and P turnover in the disturbed biocrusts (DH, DB), compared with the undisturbed biocrusts (UB, U). This corresponds to our results that the nutrient contents of tailing biocrusts, including OC, TP, NO_3_-N, and NH_4_-N were significantly lower in the disturbed biocrusts. Furthermore, the significant correlations between physicochemical properties and most enzymatic activities were found in this study (Supplementary Table 2). After 15 years of natural development (U), biocrusts significantly improved the nutritional level of tailings, fixing the tailings surface and increasing C, N, P, and other nutrient contents. However, the destruction of biocrusts, caused by physical disturbance, led to significantly decreased nutrient contents and enzyme activities in the tailings substrate, posing a potential slowdown to the subsequent ecological restoration of the mine tailings.

### Physical Disturbance Changed Structure and Composition of Biocrusts Bacteria Community

Our research results show that disturbance led to a significant shift of dominant taxa in biocrusts. Our results indicate that physical disturbance exhibits a significant negative effect on biocrusts bacterial communities in mine tailings. Specifically, Cyanobacteria was the dominant phylum in undisturbed biocrusts (U), and its relative abundance was significantly higher than other phyla; however, physical disturbance strongly decreased its relative abundance (DH, DB). This result is consistent with the changes in chlorophyll *a* and scytonemin. Chloroflexi was observed to occur in close contact with cyanobacteria in biocrusts. Burow et al. (2013) propose a metabolic pathway between *Microcoleus* spp., fermenting photosynthates to organic acids, and *Chloroflexi* spp., taking these up to be stored as polyhydroxyalkanoates (Ley et al., 2006; Burow et al., 2013). This metabolic link might be a more general phenomenon that could explain the synchronous decreased relative abundance of Chloroflexi and Cyanobacteria in the disturbed biocrusts.

The relative abundance of Acidobacteria and Actinobacteria in the disturbed biocrusts (DH, DB) was significantly higher than that in the undisturbed biocrusts (UB, U). Studies reveal that Acidobacteria and Actinobacteria are oligotrophic bacteria, which are always found in bare sand in dryland areas (Kalam et al., 2020). The relative abundance of Acidobacteria is found to decrease with the increase of C content, which is negatively correlated with the mineralization rate of the soil (Pascault et al., 2013). This may help explain our low nutrient level of DH (OC, TN, TP, NO_3_-N, and NH_4_-N) corresponding to the high relative abundance of Acidobacteria and Actinobacteria.

At the genus level, the relative abundance of *Microcoleus* in the undisturbed biocrusts (U) was as high as 24.7%, much higher than other genera, whereas it was lower than 1% in the disturbed biocrusts (DH), and its dominance was replaced by *Thermoanaerobacterium*. *Microcoleus* was the general dominant genus of photosynthetic autotrophs in early biocrusts globally (Garcia-Pichel and Wojciechowski, 2009), playing an essential role in organic carbon input and biocrust formation in global drylands (Wang et al., 2020). After physical disturbance, it was completely replaced by *Thermoanaerobacterium*, which was sporulated in biomes in low-nutrient environments, such as dry soil or tailings (Maier et al., 2018). It is reported that *Thermoanaerobacterium* have strong resistance against UV light, heavy metals, and oxidative stress (Rosenberg et al., 2014), thus the highest relative abundance of *Thermoanaerobacterium* in the present study was found in DH. Different from the biocrusts in drylands, filamentous bacterium *Kouleothrix*, the main filamentous bacteria for sludge bulking in sewage treatment plants (Nittami et al., 2019, 2020), was also found in this study, and this may be ascribed to the geographical location of this gold mine.

Generally, physical disturbance caused significant changes in the microbial community at both the phylum and genus levels. The dominance of photosynthetic autotrophs (Cyanobacteria) in undisturbed biocrusts was replaced by heterotrophic bacteria (Proteobacteria and Actinobacteria), and the relative abundance of oligotrophic species, such as Acidobacteria and Actinobacteria increased significantly after physical disturbance. This change implies a significant impact on the carbon and nitrogen metabolism and circulation of biocrusts (Morillas and Gallardo, 2015). Biocrusts play a key role in terrestrial carbon input through photosynthesis, especially in drylands, tailings ponds, and other oligotrophic ecosystems; lacking high vegetation, biocrusts are considered as the main or even the only source of carbon sequestration in these areas (Li et al., 2012). However, physical disturbance significantly reduces the photosynthetic biomass and nutrient contents of biocrusts (Faist et al., 2017). In this study, our findings also suggest that the control of physical disturbance is extremely important for maintaining ecological function of biocrusts. In addition, although in most cases biocrusts can develop naturally in the disturbed tailings, bioremediation projects are expected to accelerate this process. Based on our results, cyanobacterial inoculation (e.g., *Microcoleus*) is expected to serve as an option to induce biocrusts in the disturbed tailings, which is proven with high feasibility in dryland restoration (Lan et al., 2014b), and has the potential to transfer nutrients from domestic wastewater to the soils of tailings (Wu et al., 2018).

### Physical Disturbance Changed the Interaction of Bacterial Community in Biocrusts

Physical disturbance not only depressed the photosynthetic biomass and changed the bacterial community structure, but it also changed the keystone taxa and interactions of the bacterial community. In the UB and U network, cyanobacteria was the keystone phylum (Figures 6C,D). As the photoautotrophic organism, cyanobacteria controlled the abundance, diversity, and physiology of heterotrophic organisms (Maier et al., 2018), and a symbiotic nutrient exchange was proposed within the “cyanosphere” (Nelson et al., 2021). However, in the networks of disturbed biocrusts (DH, DB), Proteobacteria phylum was identified as the keystone taxa (Figures 6A,B), playing the key role in the bacterial interactions. In physical crusts and the early stage of biocrusts, Proteobacteria was considered to play an important role in resisting wind erosion and nitrogen fixation (Gundlapally and Garcia-Pichel, 2006), speeding up the formation of biocrusts (Zhou et al., 2020).

Changes of the network topological structure demonstrate that the bacterial interactions became more intricate and strengthened after disturbance. This is shown by (i) comparing with the undisturbed biocrusts (UB, U), an obvious increase in the number of edges in bacterial networks after physical disturbance (DH, DB; Table 3); (ii) in DH and DB, networks became more clustered (Table 3). In addition, a group of taxa that have a common phylogeny and/or similar ecological niche or have potential interactions was defined as a module hub. Module hubs in the different networks were expected to be distributed in different taxa. Specifically, module hubs in the DH network were Proteobacteria (31.67%), Actinobacteria (22.5%), and Chloroflexi (12.5%); module hubs in the U network were Proteobacteria (22.83%), Chloroflexi (19.57%), and Cyanobacteria (18.48%). A more complicated network structure in DH could be attributed to the destruction of better developed biocrusts, providing a “blank” environment for more bacteria to colonize. Under this circumstance, Cyanobacteria play an important role in maintaining the stability of bacterial communities in U. Modularity is an indicator that characterizes the stability of the network. The network analysis demonstrates that physical disturbance reduces the modularity of the overall bacterial network. Some studies propose that positive edges are deemed to be unstable in the community structure, and members of the community may respond in tandem to physical disturbance, resulting in positive feedback and co-oscillation (de Vries et al., 2018). Both the modularity and negative correlation in the network increase the stability of the network under disturbances (Coyte et al., 2015). In this study, a much lower proportion of negative edges (about 18.8%) was found in the disturbed biocrusts, indicating an unstable bacterial community caused by physical disturbance, and this result may be ascribed to the dramatic decrease of OC and photosynthetic biomass in the disturbed biocrusts.

## Conclusion

In this study, the impact of physical disturbance on physicochemical properties, bacterial community structure, and ecological functions of biocrusts in a gold mine tailing in central China was studied. The results fill the gaps in the improvement of biocrusts on a substrate and the effects of physical disturbance on biocrusts in mine tailings of a humid area. Our results show that biocrusts could reform on the substrate surface after physical disturbance; however, the physical disturbance strongly decreased its nutrient contents (NO_3_-N, NH_4_-N, OC, and TP) and enzyme activities (urease, nitrogenase, neutral phosphatase, and sucrase) and changed its bacterial community structure. Additionally, the dominant taxa of biocrusts (e.g., *Microcoleus*) in mine tailings were the same as those from dryland biocrusts; however, the physical disturbance caused their dominance to be replaced by heterotrophic bacteria. Overall, our results demonstrate the potential of biocrusts in improving the physiochemical properties of mine tailing substrates, and based on our study, we present the future work of artificial construction of biocrusts through cyanobacteria inoculation (e.g., the dominant *Microcoleus* found in the present study), the technology of which is proven with high feasibility in dryland restoration, onto mine tailing substrate to achieve mine tailings restoration.

## Data Availability Statement

The datasets presented in this study can be found in online repositories. The names of the repository/repositories and accession number(s) can be found below: https://www.ncbi.nlm.nih.gov/, SRP 297388.

## Author Contributions

JX: experiments design, performing, and original draft writing. ZZ: experiments design. LY and LX: data analysis. LQ: performing part of the experiments. SS: experimental guidance. MF: technical checking of the manuscript. RT: writing and editing. SL: data visualization. LW: manuscript conceptualization and experimental design. All authors contributed to the article and approved the submitted version.

## Conflict of Interest

The authors declare that the research was conducted in the absence of any commercial or financial relationships that could be construed as a potential conflict of interest.

## Publisher’s Note

All claims expressed in this article are solely those of the authors and do not necessarily represent those of their affiliated organizations, or those of the publisher, the editors and the reviewers. Any product that may be evaluated in this article, or claim that may be made by its manufacturer, is not guaranteed or endorsed by the publisher.

## Footnotes

1
http://rdp.cme.msu.edu/

2
https://gephi.org/

## References

[B1] Alsharif W. Saad M. M. Hirt H. (2020). Desert microbes for boosting sustainable agriculture in extreme environments. *Front. Microbiol.* 11:1666. 10.3389/fmicb.2020.01666 32793155 PMC7387410

[B2] Belnap J. (1995). Surface disturbances: their role in accelerating desertification. *Environ. Monit. Assess.* 37 39–57. 24197839 10.1007/BF00546879

[B3] Belnap J. (2003). The world at your feet: desert biological soil crusts. *Front. Ecol. Environ.* 1 181–189. 10.1890/1540-92952003001[0181:TWAYFD]2.0.CO;2

[B4] Belnap J. (2006). The potential roles of biological soil crusts in dryland hydrologic cycles. *Hydrol. Process.* 20 3159–3178. 10.1002/hyp.6325

[B5] Belnap J. Büdel B. (2016). “Biological soil crusts as soil stabilizers,” in *Biological Soil Crusts: An Organizing Principle in Drylands*, eds Weber B. Büdel B. Belnap J., (Berlin: Springer), 305–320.

[B6] Belnap J. Büdel B. Lange O. L. (2001). “Biological soil crusts: characteristics and distribution,” in *Biological Soil Crusts: Structure, Function, and Management. Ecological Studies (Analysis and Synthesis)*, eds Belnap J. Lange O. L, (Berlin: Springer).

[B7] Bowker M. A. Belnap J. Büdel B. Sannier C. Rivera-Aguilar V. (2016). *Controls on Distribution Patterns of Biological Soil Crusts at Micro- to Global Scales.* Berlin: Springer International Publishing.

[B8] Burow L. C. Woebken D. Marshall I. P. G. Lindquist E. A. Bebout B. M. Prufert-Bebout L. (2013). Anoxic carbon flux in photosynthetic microbial mats as revealed by metatranscriptomics. *ISME J.* 7 817–829. 10.1038/ismej.2012.150 23190731 PMC3603402

[B9] Cabala J. Rahmonov O. Jablonska M. Teper E. (2011). Soil algal colonization and its ecological role in an environment polluted by past Zn-Pb mining and smelting activity. *Water Air Soil Pollut.* 215 339–348. 10.1007/s11270-010-0482-1

[B10] Cerna B. Rejmankova E. Snyder J. M. Santruckova H. (2009). Heterotrophic nitrogen fixation in oligotrophic tropical marshes: changes after phosphorus addition. *Hydrobiologia* 627 55–65. 10.1007/s10750-009-9715-y

[B11] Chen L. Rossi F. Deng S. Liu Y. Wang G. Adessi A. (2014). Macromolecular and chemical features of the excreted extracellular polysaccharides in induced biological soil crusts of different ages. *Soil Biol. Biochem.* 78 1–9. 10.1016/j.soilbio.2014.07.004

[B12] Colica G. Li H. Rossi F. Li D. Liu Y. De Philippis R. (2014). Microbial secreted exopolysaccharides affect the hydrological behavior of induced biological soil crusts in desert sandy soils. *Soil Biol. Biochem.* 68, 62–70.

[B13] Coyte K. Z. Schluter J. Foster K. R. (2015). The ecology of the microbiome: networks, competition, and stability. *Science* 350 663–666. 10.1126/science.aad2602 26542567

[B14] Darby B. J. Neher D. A. Belnap J. (2010). Impact of biological soil crusts and desert plants on soil microfaunal community composition. *Plant Soil* 328 421–431.

[B15] de Vries F. T. Griffiths R. I. Bailey M. Craig H. Girlanda M. Gweon H. S. (2018). Soil bacterial networks are less stable under drought than fungal networks. *Nat. Commun.* 9:3033 10.1038/s41467-018-05516-7 30072764 PMC6072794

[B16] Doane T. A. Horwath W. R. (2003). Spectrophotometric determination of nitrate with a single reagent. *Anal. Lett.* 36 2713–2722. 10.1081/al-120024647

[B17] Dojani S. Büdel B. Deutschewitz K. Weber B. (2011). Rapid succession of biological soil crusts after experimental disturbance in the Succulent Karoo, South Africa. *Appl. Soil Ecol.* 48 263–269.

[B18] Dubois M. Gilles K. A. Hamilton J. K. Rebers P. T. Smith F. (1956). Colorimetric method for determination of sugars and related substances. *Anal. Chem.* 28 350–356.

[B19] Faist A. M. Herrick J. E. Belnap J. Van Zee J. W. Barger N. N. (2017). Biological soil crust and disturbance controls on surface hydrology in a semi-arid ecosystem. *Ecosphere* 8:13. 10.1002/ecs2.1691

[B20] Ferrenberg S. Reed S. C. Belnap J. (2015). Climate change and physical disturbance cause similar community shifts in biological soil crusts. *Proc. Natl. Acad. Sci. U.S.A.* 112 12116–12121. 10.1073/pnas.1509150112 26371310 PMC4593113

[B21] Fick S. E. Day N. Duniway M. C. Hoy-Skubik S. Barger N. N. (2020). Microsite enhancements for soil stabilization and rapid biocrust colonization in degraded drylands. *Restor. Ecol.* 28 S139–S149. 10.1111/rec.13071

[B22] Gao L. Q. Bowker M. A. Sun H. Zhao J. Zhao Y. G. (2020). Linkages between biocrust development and water erosion and implications for erosion model implementation. *Geoderma* 357:9. 10.1016/j.geoderma.2019.113973

[B23] Garcia-Pichel F. Wojciechowski M. F. (2009). The evolution of a capacity to build supra-cellular ropes enabled filamentous cyanobacteria to colonize highly erodible substrates. *PLoS One* 4:7801. 10.1371/journal.pone.0007801 19924246 PMC2773439

[B24] Garcia-Pichel F. Castenholz R. W. (1991). Characterization and biological implications of scytonemin, a cyanobacterial sheath pigment 1. *J. Phycol.* 27 395–409.

[B25] Gundlapally S. R. Garcia-Pichel F. (2006). The community and phylogenetic diversity of biological soil crusts in the Colorado Plateau studied by molecular fingerprinting and intensive cultivation. *Microb. Ecol.* 52 345–357. 10.1007/s00248-006-9011-6 16691327

[B26] Gypser S. Herppich W. B. Fischer T. Lange P. Veste M. (2016). Photosynthetic characteristics and their spatial variance on biological soil crusts covering initial soils of post-mining sites in Lower Lusatia, NE Germany. *Flora* 220 103–116. 10.1016/j.flora.2016.02.012

[B27] Hagemann M. Henneberg M. Felde V. J. M. N. L. Berkowicz S. M. Raanan H. Pade N. (2017). Cyanobacterial populations in biological soil crusts of the northwest Negev Desert, Israel-effects of local conditions and disturbance. *FEMS Microbiol. Ecol.* 93 1–9. 10.1093/femsec/fiw228 27810874

[B28] He M. Hu R. Jia R. (2019). Biological soil crusts enhance the recovery of nutrient levels of surface dune soil in arid desert regions. *Ecol. Indic.* 106:105497.

[B29] Huang L.-N. Tang F.-Z. Song Y.-S. Wan C.-Y. Wang S.-L. Liu W.-Q. (2011). Biodiversity, abundance, and activity of nitrogen-fixing bacteria during primary succession on a copper mine tailings. *FEMS Microbiol. Ecol.* 78 439–450. 10.1111/j.1574-6941.2011.01178.x 22066852

[B30] Huang J. Yu H. Guan X. Wang G. Guo R. (2016). Accelerated dryland expansion under climate change. *Nat. Clim. Change* 6:166-+. 10.1038/nclimate2837

[B31] Jiang Z.-Y. Li X.-Y. Wei J.-Q. Chen H.-Y. Li Z.-C. Liu L. (2018). Contrasting surface soil hydrology regulated by biological and physical soil crusts for patchy grass in the high-altitude alpine steppe ecosystem. *Geoderma* 326 201–209. 10.1016/j.geoderma.2018.04.009

[B32] Kakeh J. Gorji M. Mohammadi M. H. Asadi H. Khormali F. Sohrabi M. (2020). Biological soil crusts determine soil properties and salt dynamics under arid climatic condition in Qara Qir, Iran. *Sci. Total Environ.* 732:139168. 10.1016/j.scitotenv.2020.139168 32442768

[B33] Kakeh J. Gorji M. Mohammadi M. H. Asadi H. Khormali F. Sohrabi M. (2021). Biocrust islands enhance infiltration, and reduce runoff and sediment yield on a heavily salinized dryland soil. *Geoderma* 404:115329 10.1016/j.geoderma.2021.115329

[B34] Kalam S. Basu A. Ahmad I. Sayyed R. El Enshasy H. A. Dailin D. J. (2020). Recent understanding of soil Acidobacteria and their ecological significance: a critical review. *Front. Microbiol.* 11:580024. 10.3389/fmicb.2020.580024 33193209 PMC7661733

[B35] Lafuente A. Durán J. Delgado-Baquerizo M. Recio J. Gallardo A. Singh B. K. (2020). Biocrusts modulate responses of nitrous oxide and methane soil fluxes to simulated climate change in a mediterranean dryland. *Ecosystems* 23 1690–1701.

[B36] Lan S. Ouyang H. Wu L. Zhang D. Hu C. (2017). Biological soil crust community types differ in photosynthetic pigment composition, fluorescence and carbon fixation in Shapotou region of China. *Appl. Soil Ecol.* 111 9–16. 10.1016/j.apsoil.2016.11.009

[B37] Lan S. Wu L. Zhang D. Hu C. (2013). Assessing level of development and successional stages in biological soil crusts with biological indicators. *Microb. Ecol.* 66 394–403. 10.1007/s00248-013-0191-6 23389251

[B38] Lan S. Wu L. Zhang D. Hu C. (2014a). Desiccation provides photosynthetic protection for crust cyanobacteria *Microcoleus vaginatus* from high temperature. *Physiol. Plant* 152 345–354. 10.1111/ppl.12176 24611508

[B39] Lan S. Wu L. Zhang D. Hu C. (2015). Analysis of environmental factors determining development and succession in biological soil crusts. *Sci. Total Environ.* 538 492–499. 26318686 10.1016/j.scitotenv.2015.08.066

[B40] Lan S. Zhang Q. Wu L. Liu Y. Zhang D. Hu C. (2014b). Artificially accelerating the reversal of desertification: cyanobacterial inoculation facilitates the succession of vegetation communities. *Environ. Sci. Technol.* 48 307–315. 10.1021/es403785j 24303976

[B41] Langhans T. M. Storm C. Schwabe A. (2010). Regeneration processes of biological soil crusts, macro-cryptogams and vascular plant species after fine-scale disturbance in a temperate region: recolonization or successional replacement? *Flora* 205 46–60. 10.1016/j.flora.2008.12.001

[B42] Levi N. Hillel N. Zaady E. Rotem G. Ziv Y. Karnieli A. (2021). Soil quality index for assessing phosphate mining restoration in a hyper-arid environment. *Ecol. Indic.* 125:13. 10.1016/j.ecolind.2021.107571

[B43] Ley R. E. Harris J. K. Wilcox J. Spear J. R. Miller S. R. Bebout B. M. (2006). Unexpected diversity and complexity of the Guerrero Negro hypersaline microbial mat. *Appl. Environ. Microbiol.* 72 3685–3695. 10.1128/aem.72.5.3685-3695.2006 16672518 PMC1472358

[B44] Li X. R. Zhang P. Su Y. G. Jia R. L. (2012). Carbon fixation by biological soil crusts following revegetation of sand dunes in arid desert regions of China: a four-year field study. *Catena* 97 119–126. 10.1016/j.catena.2012.05.009

[B45] Li Y.-G. Zhou X.-B. Zhang Y.-M. (2019). Moss patch size and microhabitats influence stoichiometry of moss crusts in a temperate desert, Central Asia. *Plant Soil* 443 55–72. 10.1007/s11104-019-04191-x

[B46] Liu W.-q. Song Y.-s. Wang B. Li J.-t. Shu W.-s. (2012). Nitrogen fixation in biotic crusts and vascular plant communities on a copper mine tailings. *Eur. J. Soil Biol.* 50 15–20. 10.1016/j.ejsobi.2011.11.009

[B47] Maier S. Tamm A. Wu D. Caesar J. Grube M. Weber B. (2018). Photoautotrophic organisms control microbial abundance, diversity, and physiology in different types of biological soil crusts. *ISME J.* 12 1032–1046. 10.1038/s41396-018-0062-8 29445133 PMC5864206

[B48] Marguí E. Queralt I. Carvalho M. Hidalgo M. (2005). Comparison of EDXRF and ICP-OES after microwave digestion for element determination in plant specimens from an abandoned mining area. *Anal. Chim. Acta* 549 197–204.

[B49] Morillas L. Gallardo A. (2015). Biological soil crusts and wetting events: effects on soil N and C cycles. *Appl. Soil Ecol.* 94 1–6. 10.1016/j.apsoil.2015.04.015

[B50] Munkhtsetseg E. Shinoda M. Ishizuka M. Mikami M. Kimura R. Nikolich G. (2017). Anthropogenic dust emissions due to livestock trampling in a Mongolian temperate grassland. *Atmos. Chem. Phys.* 17 11389–11401. 10.5194/acp-17-11389-2017

[B51] Nelson C. Giraldo-Silva A. Garcia-Pichel F. (2021). A symbiotic nutrient exchange within the cyanosphere microbiome of the biocrust cyanobacterium, *Microcoleus vaginatus*. *ISME J.* 15 282–292. 32968213 10.1038/s41396-020-00781-1 PMC7853076

[B52] Nelson D. W. (1996). Total carbon, organic carbon, and organic matter. *Methods Soil Anal.* 9 961–1010.

[B53] Nittami T. Kasakura R. Kobayashi T. Suzuki K. Koshiba Y. Fukuda J. (2020). Exploring the operating factors controlling Kouleothrix (type 1851), the dominant filamentous bacterial population, in a full-scale A2O plant. *Sci. Rep.* 10:6809. 10.1038/s41598-020-63534-2 32321952 PMC7176654

[B54] Nittami T. Shoji T. Koshiba Y. Noguchi M. Oshiki M. Kuroda M. (2019). Investigation of prospective factors that control Kouleothrix (Type 1851) filamentous bacterial abundance and their correlation with sludge settleability in full-scale wastewater treatment plants. *Process Saf. Environ. Prot.* 124 137–142. 10.1016/j.psep.2019.02.003

[B55] Nyenda T. Gwenzi W. Piyo T. T. Jacobs S. M. (2019a). Occurrence of biological crusts and their relationship with vegetation on a chronosequence of abandoned gold mine tailings. *Ecol. Eng.* 139:105559. 10.1016/j.ecoleng.2019.07.029

[B56] Nyenda T. Jacobs S. M. Gwenzi W. Muvengwi J. (2019b). Biological crusts enhance fertility and texture of gold mine tailings. *Ecol. Eng.* 135 54–60. 10.1016/j.ecoleng.2019.03.007

[B57] Papatheodorou E. M. Papapostolou A. Monokrousos N. Jones D. W. Scullion J. Stamou G. P. (2020). Crust cover and prior soil moisture status affect the response of soil microbial community and function to extreme rain events in an arid area. *Eur. J. Soil Biol.* 101:103243 10.1016/j.ejsobi.2020.103243

[B58] Pascault N. Ranjard L. Kaisermann A. Bachar D. Christen R. Terrat S. (2013). Stimulation of different functional groups of bacteria by various plant residues as a driver of soil priming effect. *Ecosystems* 16 810–822. 10.1007/s10021-013-9650-7

[B59] Potts M. (1994). Desiccation tolerance of prokaryotes. *Microbiol. Rev.* 58 755–805. 7854254 10.1128/mr.58.4.755-805.1994 PMC372989

[B60] Potts M. (1999). Mechanisms of desiccation tolerance in cyanobacteria. *Eur. J. Phycol.* 34 319–328.

[B61] Pushkareva E. Baumann K. Anh Tu V. Mikhailyuk T. Baum C. Hrynkiewicz K. (2021). Diversity of microbial phototrophs and heterotrophs in Icelandic biocrusts and their role in phosphorus-rich Andosols. *Geoderma* 386:114905 10.1016/j.geoderma.2020.114905

[B62] Remon E. Bouchardon J.-L. Cornier B. Guy B. Leclerc J.-C. Faure O. (2005). Soil characteristics, heavy metal availability and vegetation recovery at a former metallurgical landfill: implications in risk assessment and site restoration. *Environ. Pollut.* 137 316–323. 15913857 10.1016/j.envpol.2005.01.012

[B63] Rippin M. Lange S. Sausen N. Becker B. (2018). Biodiversity of biological soil crusts from the Polar Regions revealed by metabarcoding. *FEMS Microbiol. Ecol.* 94:fiy036. 10.1093/femsec/fiy036 29514253

[B64] Rodriguez-Caballero E. Belnap J. Buedel B. Crutzen P. J. Andreae M. O. Poeschl U. (2018). Dryland photoautotrophic soil surface communities endangered by global change. *Nat. Geosci.* 11 185–189. 10.1038/s41561-018-0072-1

[B65] Rosenberg E. Delong E. F. Lory S. Stackebrandt E. Thompson F. (2014). The Prokaryotes Actinobacteria. Berlin: Springer.

[B66] Rossi F. De Philippis R. (2016). “Exocellular polysaccharides in microalgae and cyanobacteria: chemical features, role and enzymes and genes involved in their biosynthesis, in *The Physiology of Microalgae*, eds Borowitzka M. A. Beardall J. Raven J. A., (Berlin: Springer International publishing), 565–590.

[B67] Rossi F. Micheletti E. Bruno L. Adhikary S. P. Albertano P. Philippis R. D. (2012). Characteristics and role of the exocellular polysaccharides produced by five cyanobacteria isolated from phototrophic biofilms growing on stone monuments. *Biofouling* 28 215–224. 10.1080/08927014.2012.663751 22352355

[B68] Rossi F. Mugnai G. De Philippis R. (2018). Complex role of the polymeric matrix in biological soil crusts. *Plant Soil* 429 19–34. 10.1007/s11104-017-3441-4

[B69] Skuji, nš J. Burns R. (1976). Extracellular enzymes in soil. *CRC Crit. Rev. Microbiol.* 4 383–421. 780056 10.3109/10408417609102304

[B70] Song Y. Shu W. Wang A. Liu W. (2014). Characters of soil algae during primary succession on copper mine dumps. *J. Soils Sediments* 14 577–583. 10.1007/s11368-013-0815-y

[B71] Steven B. Yeager C. Belnap J. Kuske C. R. (2014). Common and distinguishing features of the bacterial and fungal communities in biological soil crusts and shrub root zone soils. *Soil Biol. Biochem.* 69 302–312.

[B72] Stewart K. J. Grogan P. Coxson D. S. Siciliano S. D. (2014). Topography as a key factor driving atmospheric nitrogen exchanges in arctic terrestrial ecosystems. *Soil Biol. Biochem.* 70 96–112. 10.1016/j.soilbio.2013.12.005.

[B73] Wang H. Tang X. Wang H. Shao H.-B. (2015). Proline accumulation and metabolism-related genes expression profiles in Kosteletzkya virginica seedlings under salt stress. *Front. Plant Sci.* 6:792. 10.3389/fpls.2015.00792 26483809 PMC4586422

[B74] Wang J. Zhang P. Bao J.-T. Zhao J.-C. Song G. Yang H.-T. (2020). Comparison of cyanobacterial communities in temperate deserts: a cue for artificial inoculation of biological soil crusts. *Sci. Total Environ.* 745:140970 10.1016/j.scitotenv.2020.140970 32731072

[B75] Whistler R. L. Kirby K. W. (2002). Composition and behavior of soil polysaccharides1,2. *J. Am. Chem. Soc.* 78 1755–1759.

[B76] Wu L. Lan S. Zhang D. Hu C. (2013). Functional reactivation of photosystem II in lichen soil crusts after long-term desiccation. *Plant Soil* 369 177–186. 10.1007/s11104-012-1563-2

[B77] Wu L. Zhang G. Lan S. Zhang D. Hu C. (2014). Longitudinal photosynthetic gradient in crust lichens’ thalli. *Microb. Ecol.* 67 888–896. 24477924 10.1007/s00248-014-0366-9

[B78] Wu L. Zhu Q. Yang L. Li B. Hu C. Lan S. (2018). Nutrient transferring from wastewater to desert through artificial cultivation of desert cyanobacteria. *Bioresour. Technol.* 247 947–953. 30060434 10.1016/j.biortech.2017.09.127

[B79] Yang H. Y. Liu C. Z. Liu Y. M. Xing Z. S. (2018). Impact of human trampling on biological soil crusts determined by soil microbial biomass, enzyme activities and nematode communities in a desert ecosystem. *Eur. J. Soil Biol.* 87 61–71. 10.1016/j.ejsobi.2018.05.005

[B80] Ye Z. H. Shu W. S. Zhang Z. Q. Lan C. Y. Wong M. H. (2002). Evaluation of major constraints to revegetation of lead/zinc mine tailings using bioassay techniques. *Chemosphere* 47 1103–1111. 10.1016/s0045-6535(02)00054-1 12137044

[B81] Zhang H. Huang F. Cai G. Li Y. Lin J. (2018). Rapid and sensitive detection of *Escherichia coli* O157: H7 using coaxial channel-based DNA extraction and microfluidic PCR. *J. Dairy Sci.* 101 9736–9746. 30219420 10.3168/jds.2018-14730

[B82] Zhang L. Chen X. Xu Y. Jin M. Ye X. Gao H. (2020). Soil labile organic carbon fractions and soil enzyme activities after 10 years of continuous fertilization and wheat residue incorporation. *Sci. Rep.* 10 1–10. 32647368 10.1038/s41598-020-68163-3 PMC7347534

[B83] Zhou H. Gao Y. Jia X. Wang M. Ding J. Cheng L. (2020). Network analysis reveals the strengthening of microbial interaction in biological soil crust development in the Mu Us Sandy Land, northwestern China. *Soil Biol. Biochem.* 144:107782. 10.1016/j.soilbio.2020.107782

