# Supplementary material for: Physical Disturbance Reduces Cyanobacterial Relative Abundance and Substrate Metabolism Potential of Biological Soil Crusts on a Gold Mine Tailing of Central China
Source: Front Microbiol. 2022 Apr 6;13:811039. doi: 10.3389/fmicb.2022.811039 (PMC9019783; doi:10.3389/fmicb.2022.811039)
Supplement: Supplementary file 1 [file Data_Sheet_1.docx]

**Physical disturbance reduces cyanobacterial relative abundance and substrate metabolism potential of biological soil crusts on a gold mine tailing of Central China**

Jingshang Xiao^1^, Shubin Lan^2^, Zulin Zhang^1,3^, Lie Yang^1^, Long Qian^1^, Ling Xia^1^, Shaoxian Song^1^, María E. Farías^4^, Rosa María Torres^5^, Li Wu^1^**^[[1]](#footnote-1)^***.

*^1^ School of Resources and Environmental Engineering, Wuhan University of Technology, Wuhan, 430072, China*

*^2^Key Laboratory of Algal Biology, Institute of Hydrobiology, Chinese Academy of Sciences, Wuhan 430072, China*

*^3^ The James Hutton Institute, Craigiebuckler, Aberdeen ABI5 8QH, UK*

*^4^Laboratorio de Investigaciones Microbiológicas de Lagunas Andinas (LIMLA), Planta Piloto de Procesos Industriales Microbiológicos (PROIMI), CCT, CONICET, San Miguel de Tucumán, Tucumán, Argentina.*

*^5^CETMIC- CONICET- CCT La Plata, CICBA, Camino Centenario y 506, 1897, M. B. Gonnet, La Plata, Argentina*

**Supplementary Method**

*1.* *Illumina MiSeq sequencing data processing*

Raw fastq files were demultiplexed, quality-filtered by Trimmomatic and merged by FLASH with the following criteria: (i) The reads were truncated at any site receiving an average quality score <20 over a 50 bp sliding window. (ii) Primers were exactly matched allowing 2 nucleotide mismatching, and reads containing ambiguous bases were removed. (iii) Sequences whose overlap longer than 10 bp were merged according to their overlap sequence.

*2. Bacterial network construct*

First, the experimental data used for constructing bacterial networks were generated by pyrosequencing of 16S rDNA genes. Since the sequence numbers of individual ASVs obtained varied significantly among different samples, the relative proportions of sequence numbers were used for subsequent Spearman correlation analysis. Second, a similarity matrix was obtained by taking the absolute values of the correlation matrix. This similarity matrix measures the degree of concordance between the abundance profiles of individual ASVs across different samples. Third, an appropriate threshold for defining network structure, s_t_, is defined using the RMT-based network approach to obtain an adjacency matrix, which encodes the strength of the connection between each pair of nodes (Luo et al., 2007; Junker and Schreiber, 2011). Fourth, the submodules within a large module were detected by fast greedy modularity optimization (Clauset et al., 2004). Correlation coefficients greater than 0.5 with a corresponding of p-value less than 0.001 were considered statistically robust and were included to generate the networks.

Table S1 Biological soil crusts element composition.

|  | Al (mg/g) | Mg (mg/g) | Fe (mg/g) | Mn(mg/g) | Ti (mg/g) | Ca (mg/g) | Na (mg/g) | S (mg/g) |
| --- | --- | --- | --- | --- | --- | --- | --- | --- |
| DH | 19.84±0.45^a^ | 21.46±0.57^a^ | 82.83±15.04^b^ | 2.16±0.05^a^ | 1.05±0.05^bc^ | 180.84±4.36^a^ | 3.52±2.37^a^ | 13.15±4.24^a^ |
| DB | 18.04±0.62^b^ | 18.05±0.23^b^ | 83.18±0.23^b^ | 2.16±0.05^a^ | 1.13±0.03^b^ | 174.17±1.46^a^ | 2.45±0.38^a^ | 15.57±0.10^a^ |
| UB | 18.41±0.13^b^ | 16.13±0.11^c^ | 112.92±1.09^a^ | 2.15±0.02^a^ | 1.00±0.06^c^ | 179.36±3.83^a^ | 4.07±0.66^a^ | 11.54±0.23^a^ |
| U | 19.66±0.56^a^ | 17.28±0.71^b^ | 72.53±2.00^b^ | 1.94±0.05^b^ | 1.28±0.10^a^ | 159.71±2.35^b^ | 1.24±0.17^a^ | 11.94±0.41^a^ |

Values represent means ± standard errors (n =3). Significant differences (P < 0.05) are marked by different letters.

Table S2 Correlation between physiochemical properties and enzyme activity.

|  | S-NP | S-NPT | S-β-GC | S-α-GC | S-POD | S-SC | S-UE | S-PPO | NITS |
| --- | --- | --- | --- | --- | --- | --- | --- | --- | --- |
| TOC | 0.606^*^ | -0.467 | 0.592^*^ | 0.790^**^ | 0.569 | 0.424 | 0.736^**^ | 0.257 | 0.918^**^ |
| TK | -0.679^*^ | 0.187 | -0.536 | -0.709^**^ | -0.281 | -0.714^**^ | -0.864^**^ | -0.475 | -0.885^**^ |
| NO_3_^-^-N | 0.530 | -0.442 | 0.552 | 0.876^**^ | 0.492 | 0.451 | 0.741^**^ | 0.325 | 0.954^**^ |
| Scytonemin | 0.438 | -0.551 | 0.554 | 0.905^**^ | 0.506 | 0.330 | 0.673^*^ | 0.146 | 0.942^**^ |
| Chl-a | 0.492 | -0.458 | 0.634^*^ | 0.897^**^ | 0.512 | 0.417 | 0.727^**^ | 0.245 | 0.963^**^ |
| NH_4_^+^-N | 0.732^**^ | -0.290 | 0.603^*^ | 0.778^**^ | 0.487 | 0.686^*^ | 0.911^**^ | 0.426 | 0.954^**^ |
| EPS | 0.213 | 0.492 | 0.542 | -0.255 | -0.158 | 0.338 | 0.102 | 0.452 | -0.186 |
| EC | -0.679^*^ | 0.187 | -0.536 | -0.709^**^ | -0.281 | -0.714^**^ | -0.864^**^ | -0.475 | -0.885^**^ |
| pH | -0.811^**^ | 0.048 | -0.601^*^ | -0.569 | -0.543 | -0.782^**^ | -0.924^**^ | -0.487 | -0.810^**^ |

Analysis method using Pearson. ^*^(p＜0.05); ^**^(p＜0.01)

Clauset, A., Newman, M.E., and Moore, C. (2004). Finding community structure in very large networks. *Physical review E* 70(6)**,** 066111.

Junker, B.H., and Schreiber, F. (2011). *Analysis of biological networks.* John Wiley & Sons.

Luo, F., Yang, Y., Zhong, J., Gao, H., Khan, L., Thompson, D.K., et al. (2007). Constructing gene co-expression networks and predicting functions of unknown genes by random matrix theory. *BMC bioinformatics* 8(1)**,** 1-17.

1. * Corresponding author: Li Wu; School of Resources and Environmental Engineering, Wuhan University of Technology, Wuhan 430070, China. Email: [*wuli774@whut.edu.cn*](mailto:wuli774@whut.edu.cn); Mobile telephone is +86-15072424191. [↑](#footnote-ref-1)
